# Effect of cation substitution on bridgmanite elasticity: A key to interpret seismic anomalies in the lower mantle

**DOI:** 10.1038/srep33337

**Published:** 2016-09-19

**Authors:** Hiroshi Fukui, Akira Yoneda, Akihiko Nakatsuka, Noriyoshi Tsujino, Seiji Kamada, Eiji Ohtani, Anton Shatskiy, Naohisa Hirao, Satoshi Tsutsui, Hiroshi Uchiyama, Alfred Q. R. Baron

**Affiliations:** 1 Center for Novel Material Science under Multi-Extreme Conditions, Graduate School of Material Science, University of Hyogo, 3-2-1 Kouto, Kamigori, Hyogo 678-1297, Japan; 2 Materials Dynamics Laboratory, RIKEN SPring-8 Center, RIKEN, 1-1-1 Kouto, Sayo, Hyogo 689-5148, Japan; 3 Institute for Planetary Materials, Okayama University, 827 Yamada, Misasa, Tottori 682-0193, Japan; 4 Graduate School of Sciences and Technology for Innovation, Yamaguchi University, 2-16-1 Tokiwadai, Ube, Yamaguchi 755-8611, Japan; 5 Graduate School of Science, Tohoku University, 6-3 Aramaki, Aoba, Sendai, Miyagi 980-8578, Japan; 6 Frontier Research Institute for Interdisciplinary Sciences, Tohoku University, 6-3 Aramaki, Aoba, Sendai, Miyagi 980-8578, Japan; 7 V. S. Sobolev Institute of Geology and Mineralogy, Siberian Branch, Russian Academy of Sciences, Novosibirsk 630090, Russia; 8 Research and Utilization Division, Japan Synchrotron Radiation Research Institute, SPring-8, 1-1-1 Kouto, Sayo, Hyogo 689-5198, Japan

## Abstract

Seismological observations show that, in some regions of the lower mantle, an increase in bulk sound velocity, interestingly, occurs in the same volume where there is a decrease in shear velocity. We show that this anti-correlated behavior occurs on cation substitution in bridgmanite by making single crystal elasticity measurements of MgSiO_3_ and (Mg,Fe,Al)(Si,Al)O_3_ using inelastic x-ray scattering in the ambient conditions. Cation substitution of ferrous iron and aluminum may explain large low shear velocity provinces in the lower mantle.

Bridgmanite, or, *Pbnm*-type magnesium-silicate perovskite, is the dominant mineral in the Earth’s lower mantle. Materials with perovskite or related structures also attract broad attention since they can display novel physical properties such as colossal magnetoresistance^1^, multiferroicity^2^, and high-temperature superconductivity^3^. At pressures over 125 GPa (corresponding to depths more than ~2700 km) and at temperature greater than 2500 K, bridgmanite transforms to a post-perovskite (pPv) phase^4^ with the *Cmcm*-type CaIrO_3_ structure. It is widely believed that pPv is the main component of the D″ layer at the bottom of the lower mantle, which is 200 km thick just above the core mantle boundary (~2900 km depth).

In the deep mantle, between 2000 and 2891 km in depth, some regions show an increase in bulk sound velocity (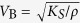), and a decrease in shear wave velocity (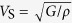): Δ*V*_B_ > 0 > Δ*V*_S_, and others show a decrease in *V*_B_ and an increase in *V*_S_: Δ*V*_B_ < 0 < Δ*V*_S_^5^,^6^ (*K*_S_, *G*, and *ρ* are adiabatic bulk modulus, shear modulus, and density, respectively). This feature is called an anti-correlated seismic velocity anomaly. It is reported that the phase transformation of (Mg,Fe,Al)(Si,Al)O_3_ from *Pbnm*-type to *Cmcm*-type can explain the increase in *V*_S_ and decrease in *V*_B_ from the average (Δ*V*_B_ < 0 < Δ*V*_S_) in some deeper regions^7^. However, this cannot explain the anomaly in the shallower part of the mantle where the pPv phase is not stable. More importantly, it is difficult to interpret the anti-correlated nature of the anomaly where Δ*V*_B_ and Δ*V*_S_ have opposite signs. The regions showing this anomaly, which are beneath Africa and the central Pacific, attract attention as large low shear velocity provinces (LLSVPs).

The origin of the LLSVPs is under debate. Thermal heterogeneity has been considered^8^, but exclusively thermal effects are insufficient to explain the LLSVPs because usually both *V*_B_ and *V*_S_ decrease with temperature. It is thus suggested that the LLSVPs have very different chemical composition from that of the average mantle^9^ due to accumulations of subducted oceanic slabs^10^, remnants of Earth’s early magma ocean^11^, or even chemical reactions with the core^12^. Recently primordial metallic melt trapped in the mantle was suggested as the nature of LLSVPs^13^. A complicated model^2^, including multiple chemical and thermal effects, can reproduce the distribution of the LLSVPs. But this model requires rather a specific distribution of effects that are not internally well correlated. Houser^14^ suggested that slow *V*_S_ might be correlated with temperature and chemical anomaly using the parameter set for bridgmanite^15^, as was used in ref. 6, but did not discuss the anti-correlated anomaly between *V*_B_ and *V*_S_. The theoretical result^15^ used in both seismological studies^6^,^14^ shows anti-correlation only in elastic moduli but not in velocities, and, more importantly, has not yet been experimentally verified.

In order to address these issues, we investigated the elastic properties of single-crystal bridgmanite at ambient conditions. Although Brillouin light scattering (BLS) is frequently used to determine elastic properties of high-pressure minerals, the elasticity of iron-bearing bridgmanite has not been determined by BLS due to its opacity, and its instability against strong optical laser irradiation. We used inelastic x-ray scattering (IXS) technique in this study. We prepared two types of bridgmanite: MgSiO_3_ (Mg-Bdg, hereafter) and Mg_0.943_Fe_0.045_Al_0.023_Si_0.988_O_3_ ((Fe,Al)-Bdg hereafter). Iron in (Fe,Al)-Bdg was confirmed by synchrotron Mössbauer spectroscopy to be in high-spin ferrous state and to occupy a large A site of perovskite structure. The sample characterization and more details of the IXS measurements are given in the Methods section. Elastic stiffness tensors (*C*_ij_) for Mg-Bdg, and for (Fe,Al)-Bdg was obtained from analysis of IXS spectra based on Christoffel’s equation^16^. A typical set of IXS spectra is shown in Fig. 1. The elastic moduli obtained are listed in Table 1 together with literature values^15^,^17^,^18^,^19^,^20^,^21^,^22^,^23^. The velocity surface plots of Mg-Bdg from the present *C*_ij_ determined from two sets of IXS measurements are shown in Fig. 2 together with those calculated from BLS results^17^,^18^,^19^. The patterns of the velocity surfaces are similar to each other: the longitudinal velocity is the fastest along about *b* axis and minimum along *c* axis, etc. The absolute values determined using IXS are generally smaller than those from BLS.

The pattern of the velocity surface of (Fe,Al)-Bdg is basically similar to that of Mg-Bdg (red and blue lines in Fig. 2). The present cation substitution affected the velocity surface as follows: 1. *V*_P_ along the *b* and *c* axes is increased; 2. the average *V*_S_ along the *b* axis is decreased; 3. the difference of *V*_S_ along the *a* and *c* axes is increased and decreased, respectively. Crystallographic studies^24^ report that iron substitution enlarges the *a* axis more than other axes, which is consistent with the present result (see Method section). The large elongation of the *a* axis probably results in the least change in *V*_P_ along *a* axis. Thus qualitatively the velocity surfaces indicate that elastic anisotropy in bridgmanite increases with the present cation substitution. More quantitatively, the acoustic anisotropy defined by 2 × (*V*_max_ − *V*_min_)/(*V*_max_ + *V*_min_) increases from 8.81% to 8.92% for longitudinal waves, and from 12.4% to 13.4% for transverse waves. The present results experimentally demonstrate that the degree of anisotropy is increased by the present cation substitution.

The Voigt-Ruess-Hill average of bulk and shear moduli calculated from *C*_ij_ are listed in Table 1. We determine *K*_S_ and *G* to be 236 and 166 GPa, respectively for Mg-Bdg. The value of *K*_S_ in the present study is consistent with that determined from RUS^20^ and that by a calculational study^22^, but is lower than the other values by ~15 GPa (6%). *G* is also smaller by ~10 GPa (also 6%) than those in the previous results. These differences correspond to 3% in velocity. The origin of the differences in *K*_S_ and *G* between two techniques should be further investigated. Nevertheless, this study experimentally demonstrated that the present cation substitution in bridgmanite increases *K*_S_ and *V*_B_ and decreases *G* and *V*_S_: an anti-correlated behavior.

This anti-correlated behavior in elastic moduli and velocities by cation substitution has not been reported. Bulk and shear moduli and velocities are summarized in Fig. 3 together with previous results. Previously, the effect of Fe substitution was investigated using ultrasonic interferometry^21^ (UI) and calculations^15^,^22^. The sample used in the UI study contained not only Fe^2+^ but also Fe^3+^. The results of the UI study disagree with one calculation^15^, where Fe^2+^ substituted for Mg^2+^, but rather agree with another^22^, where Fe^3+^ and Al^3+^ substituted for Mg^2+^ and Si^4+^. These results^21^,^22^ imply that Fe^3+^ substituting for Mg^2+^ degreases both *K*_S_ and *G*. The effect of aluminum substitution was reported using BLS^25^ and theoretical calculation^21^. An experimental study^25^ reported that the substitution of only Al decreases both elastic moduli and slightly increases *V*_B_. A theoretical study^22^ showed that the substitution of only Al decrease *V*_B_ and *V*_S_ as well as *K*_S_ and *G*. That study^22^ also investigated the effect of coupled substitution of Fe^3+^ and Al, demonstrating that the effect of this pair substitution is qualitatively the same as that of the substitution of aluminum only.

Water content sometimes reduces elastic moduli. The present samples contain a certain amount of water (140 and 460 ppm). However, it is not known how much water content affects the elasticity of bridgmanite. If water content decreased shear modulus for bridgmanite, e.g. by 0.3 GPa/100 ppm or shear velocity by 0.02 km/s/100 ppm, the present cation substitution for dry bridgmanite would show a positive correlated behavior, or increase both *K*_S_ and *G*. The anti-correlated behavior observed in this study may be due to a combination of the cation substitution and the water content.

We simply consider the effect of iron and aluminum separately thought these substitutions may be coupled. Many investigations have been done about the effect of cation substitution on isothermal bulk modulus of bridgmanite by measuring compression curves. It is well known that *K*_T0_ and *K*′ derived from a compression-curve fitting are strongly correlated. *K*_T0_ also depends on the pressure range of the measurement, sample conditions, etc. Nevertheless, the relative change in *K*_T0_ determined by the same technique is reliable. The effect of a small amount of Fe^2+^ substitution on *K*_T0_ is reported to be positive^26^,^27^,^28^,^29^. This is qualitatively consistent with the theoretical study^15^. In contrast, the effect of aluminum on *K*_T0_ is still controversial; a positive effect (increasing *K*_T0_) is reported in some studies^26^,^30^ and negative effect (decreasing *K*_T0_) in others^30^,^31^,^32^. Based on the BLS studies^17^,^18^,^19^,^25^ and the theoretical one^21^, the effect of aluminum substitution on *K*_S_ can be considered negative. Note that the theoretical study^22^ also investigated the effect of coupling substitution of Fe^3+^ and Al, demonstrating that the effect of this pair substitution is qualitatively the same as that of the substitution of aluminum only. The effect of only Fe^2+^ on the velocities can be calculated from the present study by subtracting the effect of Al from the BLS results^17^,^18^,^19^,^25^, assuming that the effects of Fe^2+^ and Al are independent. This analysis suggests that Fe^2+^ substitution increases both *V*_B_ and *V*_S_ (Table 2).

We apply the present results to a geochemical and geothermal model to estimate if this effect is sufficient to explain the LLSVPs. We assume a perovskitic lower mantle^33^ for simplicity. The seismic anomaly observed in the LLSVPs (+1 and −1% of Δ*V*_B_/*V*_B_ and Δ*V*_S_/*V*_S_, respectively)^5^,^6^ may, then be explained by variation of Fe^2+^ and Al substitution into bridgmanite at temperature conditions for 2000–2891 km depth (2250–2450 K^34^). The temperature effects on *V*_B_ and *V*_S_ were assumed to be independent of pressure and composition (Table 2). The observed anomaly of +/−1% for *V*_B_ and −/+1% for *V*_S_ corresponds to the compositional variation between MgSiO_3_ and Mg_0.959_Fe_0.027_Al_0.028_Si_0.986_O_3_, +/−2.0 atom% of ΔFe/(Mg + Fe + Si + Al) and +/−1.3 atom% of ΔAl/(Mg + Fe + Si + Al) in temperature range of 2250–2450 K^34^. This compositional heterogeneity of bridgmanite then explains the anti-correlated seismic anomaly (Figs 4 and 5AB). This model indicates that cation substitution of a few atomic percent causes an anti-correlated anomaly comparable to that observed in the LSSVPs.

We now consider to include the effect of temperature since the LLSVPs may correlate with local temperature changes. We assume the temperature difference Δ*T* between the regions with the highest *V*_B_ and the average value, i.e. Δ*T* = *T*(Δ*V*_B_/*V*_B_ = 1%) −*T*(Δ*V*_B_/*V*_B_ = 0%). The chemical inhomogeneity, ΔX/(Mg + Fe + Si + Al) (X = Fe or Al), needed to explain the velocity anomaly is then shown in Fig. 4. Especially when Δ*T* is about 113 K, the LSSVPs can be explained by only 2.7 atom% of Fe^2+^ substitution without Al variation (Fig. 5AC). More detailed modeling requires ferropericlase and taking the effect of spin transition for these two materials into account.

We have experimentally demonstrated that cation substitutions in bridgmanite enhances elastic anisotropy and causes anti-correlated behavior in elastic wave velocities. This result indicates that seismic anomalies observed in the lower mantle could be explained by chemical heterogeneity in bridgmanite.

## Methods

### Sample synthesis and characterizations

The single crystals examined in this study were synthesized at 24 GPa and 1500 °C using a Kawai-type multi anvil press (USSA-5000) installed at ISEI, Okayama University^35^. The isotope ratios of chemical reagents were at natural abundance.

The chemical compositions are confirmed as MgSiO_3_ and Mg_0.943_Fe_0.045_Al_0.023_Si_0.988_O_3_ by an electron microprobe analyzer. The number ratios of Fe/(Mg + Fe + Si + Al) and Al/(Mg + Fe + Al + Si) of this sample are 0.023 and 0.012, respectively. Assuming all Fe is bivalent, the sum of the charge estimated from the EPMA results is −0.003. This is negligible, taking the uncertainty of the chemical analysis into account. A typical amount of water content of single crystals in the run product was 140 ± 52 and 460 ± 45 ppm according to synchrotron IR absorption analyses^35^.

The Fe^3+^/ΣFe ratio of (Fe,Al)-Bdg was evaluated with synchrotron Mössbauer spectroscopy at BL10XU of SPring-8^36^. An obtained spectrum were analyzed using program MossA^37^. Without any prejudice, the spectrum seems to consist of two absorption lines with different intensities (Fig. 6). They can be interpreted either as two singlets, as an asymmetric doublet, or as combination of a doublet and a singlet. If the spectrum consists of two singlets, an isomer shift of 1.96(9) mm/s corresponds to that of monovalent high-spin iron. Considering the charge neutrality of the system, it is difficult for Fe^+^ to substitute for Mg or Si in perovskite structure. Therefore, the absorption line at 1.96 mm is the higher velocity one of a doublet. Analysis based on an asymmetric doublet gives the isomer shift of 1.05(6) mm/s and quadrupole splitting of 1.8(1) mm/s. These values indicate that iron in this sample was in a divalent high-spin state^38^ and substitute for magnesium^39^. The higher intensity at the lower velocity side is attributed either to that the sample was a single crystal or to that iron existed in another state. The former case is more plausible than the latter due to the following reasons. 1. The linewidths determined using two singlets (0.97(16) and 0.76(30) mm/s for lower and higher velocity lines) are consistent within the fitting uncertainty; 2. The line shape of the lower velocity signal looks symmetric and additional singlet/doublet to the lower velocity signal has not improved the fitting quality at all. We off course have paid much attention to possible existence of iron in a trivalent high-spin state, which should give a doublet with an isomer shift of ~0.5 mm/s^38^ and a quadrupole splitting of 0.5–1.0 mm/s^39^. Since parameter fitting assuming two doublets (one for HS Fe^2+^ and another for HS Fe^3+^) was not converged, we were not able to detect the amount of ferric iron if existed. The asymmetric doublet is probably attributed to a certain angle between the principal electric field gradient in the Fe site and the incident X-ray beam direction because the sample was a single crystal. The intensity ratio (2.7: 1) indicates the sign of the quadrupole splitting was negative. The linewidth assuming one doublet is 0.93(13) mm/s, which is much broader than a typical energy resolution of the Mössbauer spectrometer at BL10XU (0.43 mm/s). This is perhaps due to variation of the local environment around Fe in this sample given by Mg/Al/Si distribution in neighboring sites, hydrogen, and/or oxygen vacancy. The results of the synchrotron Mössbauer measurement conclude that most iron atoms were in a divalent high-spin state and occupied a magnesium site. Consequently, the simplest substitution model, where the iron substitutes for magnesium and aluminum substitutes for both magnesium and silicon in perovskite structure is consistent with the results of these analyses.

The investigated grains were confirmed to be single domains using a four-circle diffractometer with a laboratory x-ray source at room temperature. The lattice constants *a*, *b*, and *c* were 4.7784(3), 4.9306(4), 6.9005(8) Å and 4.787(1), 4.934(1), 6.904(1) Å for the Mg-Bdg and (Fe,Al)-Bdg, respectively. It was reported that the unit cell volume of MgSiO_3_ bridgmanite does not change even with 100 ppm water content^40^. An analytical curve drawn by fitting a linear function to literature values^19^,^20^,^21^,^24^,^26^,^27^,^28^,^30^,^31^,^32^,^41^,^42^,^43^,^44^,^45^,^46^,^47^,^48^,^49^,^50^,^51^,^52^,^53^ is shown in Fig. 7. The obtained analytical line for iron substitution is consistent with literature^29^. The unit cell volumes of Mg-Bdg is larger than the present analytical line by only 0.05%. Since these differences are comparable to the experimental error, the water content of 140 ppm seems to give a negligible effect. In contrast, the unit cell volume of the (Fe-Al)-Bdg is larger than those of the analytical lines by 0.29%. This excess volume may be explained by effect of aluminum and water content. It is known that aluminum incorporation increases the unit cell volume^26^,^30^,^31^,^32^. Estimating from the previous results, the value of 0.012 for Al/(Mg + Fe + Al + Si) makes the unit cell volume larger by 0.09%. Although the degree of water effect on the unit cell volume of magnesium silicate perovskite is uncertain, the water content of 460 ppm probably made the unit cell volume larger by 0.20%. The densities of the Mg-Bdg and (Fe,Al)-Bdg are 4103.3 and 4139.5 g/cm^3^, respectively.

### IXS measurement and data analysis

**Figure 1:**
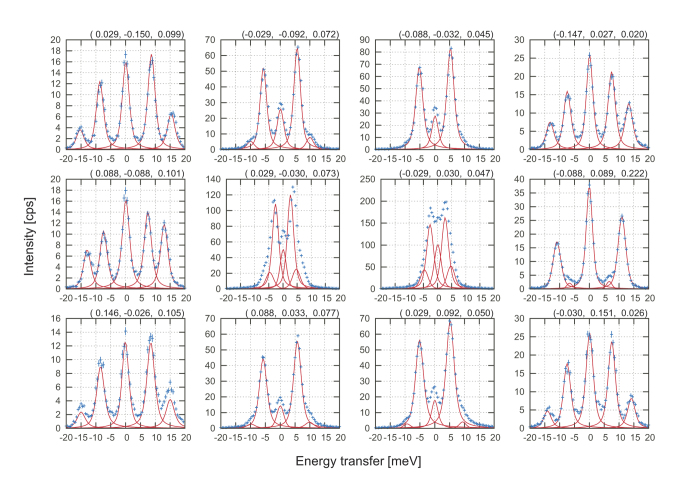
A representative set of IXS spectra of (Fe,Al)-Bdg collected at BL35XU. Twelve spectra can be measured at once. Reduced **q** positions in reciprocal lattice unit are shown in parentheses. A total momentum transfer was (2, −2, 0) + **q** in reciprocal lattice unit. Each spectrum show one or two pairs of clear phonon signals. Blue crosses show data points. Red lines show pseudo-Voight functions fitted to peaks.

**Figure 6:**
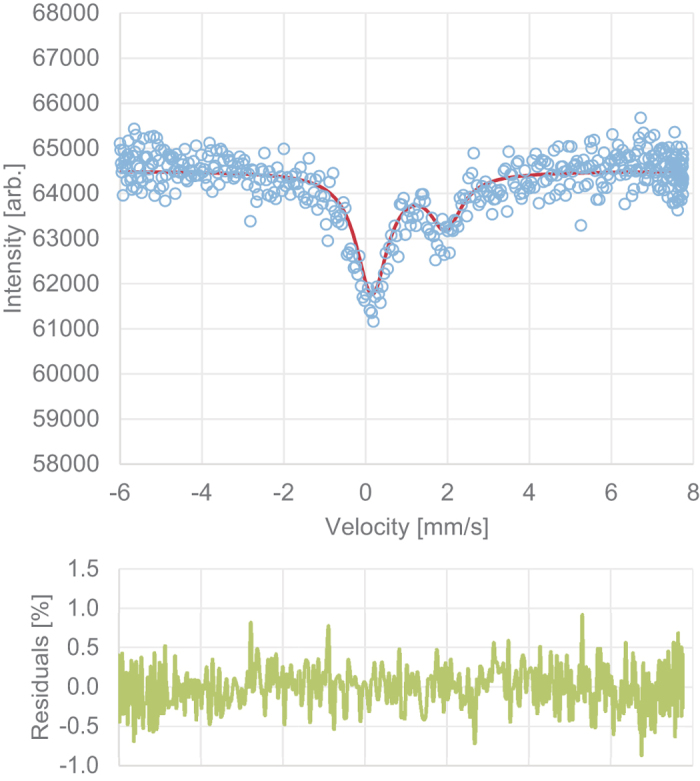
Mössbauer spectrum of ^57^Fe in (Fe,Al)-Bdg. Blue circles are raw data from which the backgrounds have been subtracted. The red and green lines indicate one doublet fitted to the data and fitting residuals. The isomer shift of 1.05 mm/s and the quadrupole splitting of 1.8 mm/s indicate that the sample contains high-spin Fe^2+^ in the magnesium site^38^,^39^.

**Figure 7:**
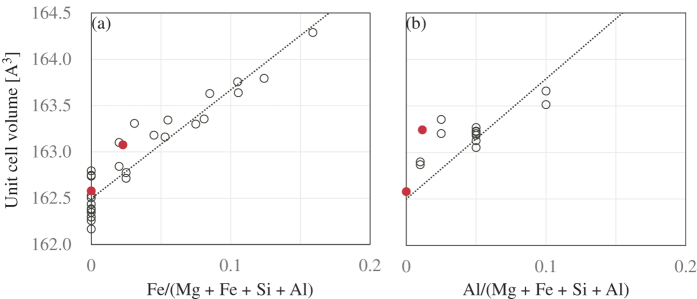
An analytical curves between unit cell volume of bridgmanite and chemical substitutions (**a**) by iron and (**b**) by aluminum. The solid (red) circles are the present results. The dotted lines were fitted to literature values shown by open cirlces^19^,^20^,^21^,^24^,^25^,^26^,^27^,^28^,^29^,^30^,^31^,^32^,^41^,^42^,^43^,^44^,^45^,^46^,^47^,^48^,^49^,^50^,^51^,^52^,^53^. (a) 11.741*x* + 162.5 and (b) 12.926*x* + 162.5, where *x* is (Fe + Al)/(Mg + Fe + Si + Al) and *V* at *x* = 0 is fixed to 162.5 Å^3^.

Inelastic X-ray scattering with a single crystal sample in conjunction with an analysis based on Christoffel’s equation has been recently used for accurate determination of elastic moduli^16^,^54^,^55^,^56^. This technique has been adopted to data along high-symmetry directions about samples at high-pressure and high-temperature conditions^55^,^56^. In this study, we did not limit data along high-symmetry directions, but measured rather redundant data at off-symmetry positions to determine *C*_ij_ precisely and to utilize all measured data with an analyzer array^16^,^54^ (see Fig. 1). We performed IXS measurements at BL35XU of SPring-8^57^ at 21.747 and 17.794 keV, with which typical energy resolutions were 1.5 and 3.0 meV full-width-half-maximum (FWHM), respectively. 21.747 keV x-ray was used for Mg-Bdg and 17.794 keV for (Fe,Al)-Bdg. The size of the incident X-ray beam was ~70 μm in diameter. We performed another measurement for Mg-Bdg to insure the quality of our results. We measured another grain from the same sample growth run at BL43LXU of SPring-8^58^. At BL43LXU, x-ray beam with size of ~20 μm and energy of 17.794 keV was used. The energy resolution was 3.0 meV (FWHM).

For each observed phonon mode, the elastic wave velocity was calculated assuming a linear relationship between phonon energy and momentum. Single crystal elasticity at ambient conditions was determined by least-square fitting to the observed velocities using the measured densities. Details of the fitting are given in ref. 16. Phonons with momentum transfers, |**q**| from 1 to 3 nm^−1^ away from Bragg peaks were used for analysis.

**Figure 8:**
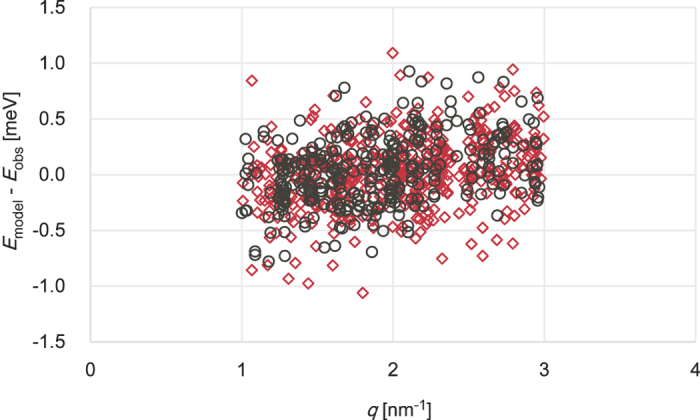
Residuals of the fitting (Δ*E* = *E*_model_ − *E*_obs_). Red diamonds and black circles are for Mg-Bdg and (Fe,Al)-Bdg, respectively.

The elastic moduli determined from data at BL35XU and BL43LXU are consistent in contrast to different BLS studies which are not so consistent. The individual results are listed in Tables 1 and 3. The different IXS measurement agree to better than 14% (the maximum deviation) whereas those from three BLS studies are spread more (26%, the maximum deviation). Therefore these two sets of IXS data for Mg-Bdg were analyzed as one set (giving 461 modes) to obtain more reliable elastic properties. For (Fe,Al)-Bdg, 319 modes were used. The residuals of the fitting are shown in Fig. 8. There is a slightly linear relationship between Δ*E* and |**q**| observed, probably meaning the assumed linear relationship between Δ*E* and |**q**| is not completely valid at this q range.

## Additional Information

**How to cite this article**: Fukui, H. *et al.* Effect of cation substitution on bridgmanite elasticity: A key to interpret seismic anomalies in the lower mantle. *Sci. Rep.* **6**, 33337; doi: 10.1038/srep33337 (2016).

## Figures and Tables

**Figure 2:**
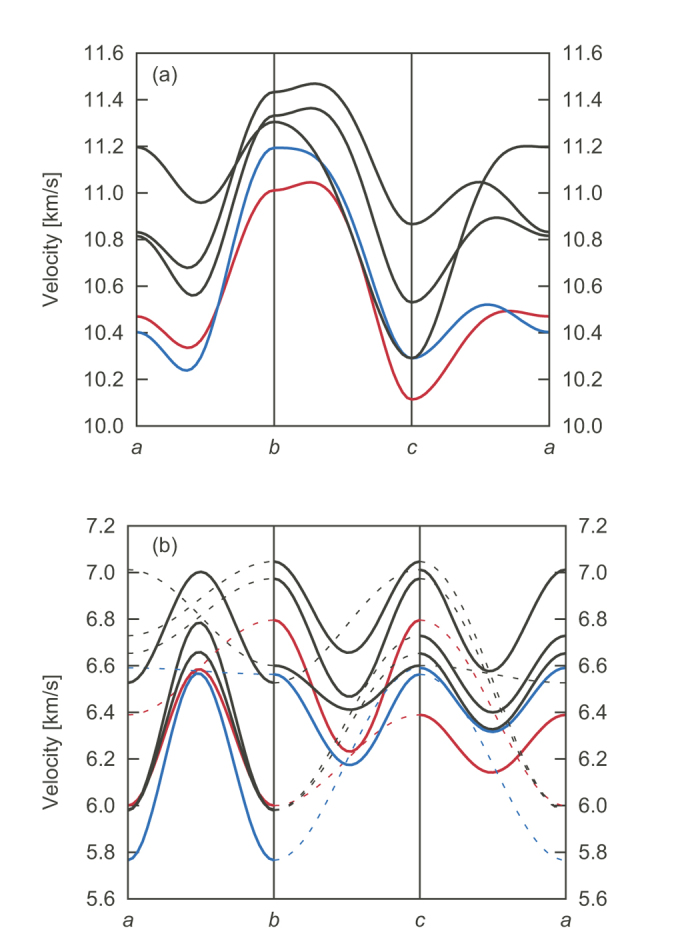
Velocity surface of bridgmanite. Longitudinal (**a**) and transverse (**b**) waves from the present study (red lines for Mg-Bdg and blue for (Fe,Al)-Bdg) and by BLS^17^,^18^,^19^ (black lines). Solid and broken lines indicate the polarization vector is in-plane or out-of-plane, respectively, in (**b**).

**Figure 3:**
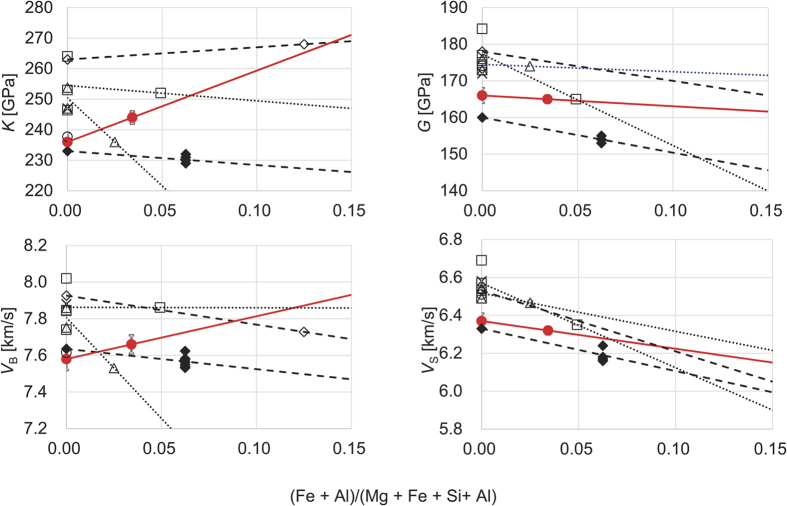
Elastic moduli and elastic velocities of bridgmanite with chemical substitution of Fe and Al for Mg and Si. The present results are shown by solid (red) circles. Open circles, squares, and triangles indicate values determined by RUS^20^, BLS^17^,^18^,^19^,^25^ and US^21^,^59^. Diamonds indicate theoretical values (open for ferrous iron (LDA)^15^ and solid for aluminum and ferric iron (GGA)^22^). Crosses indicate values by GGA-DFPT-IXS^23^. Lines are guide for eyes (solid (red): present study; dotted: previous experiments; dashed: theory).

**Figure 4:**
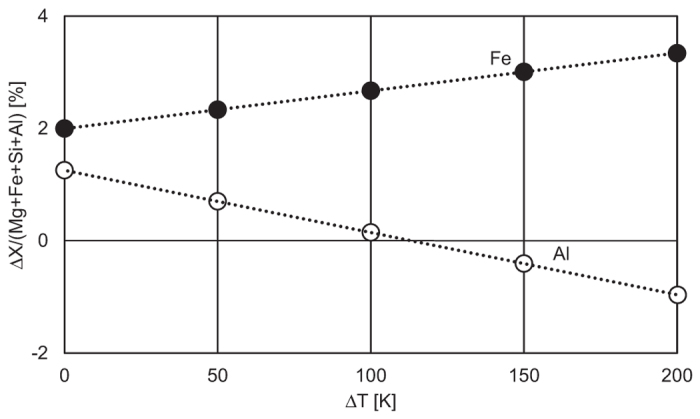
Required ΔX/(Mg + Fe + Si + Al) (X = Fe or Al) to explain the velocity anomaly of +1% for *V*_B_ and −1% for *V*_S_ with temperature difference Δ*T*. Solid and open circles are for Fe and Al, respectively. With increasing Δ*T*, required ΔFe and ΔAl increases and decreases respectively. When Δ*T* is around 110 K, the velocity anomaly can be explained only with ΔFe and the distribution of Al should be uniform.

**Figure 5:**
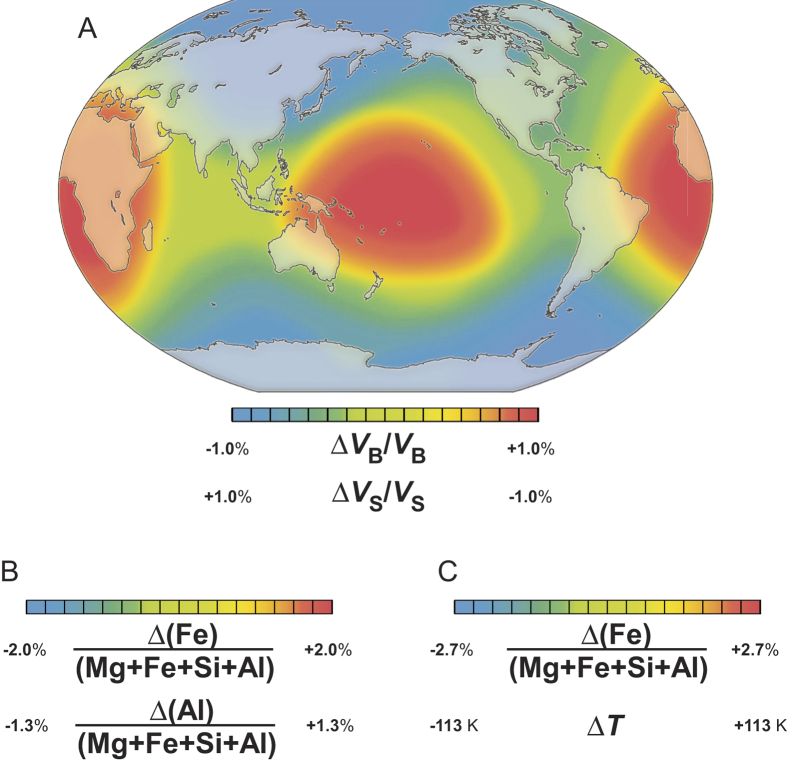
Schematic image of regional variation of seismic velocities, chemical composition of bridgmanite, and temperature variation at depth between 2000 and 2891 km. (**A**) Red color indicate the anomaly of Δ*V*_B_ > 0 > Δ*V*_S_. The map outline was made using CraftMAP (http://www.craftmap.box-i.net/). (**B**) The case of Δ*T* (difference from the average temperature) = 0. Fe and Al contents are the highest in the red regions. (**C**) The case of ΔAl/(Mg + Fe + Si + Al) = 0. Fe content is the highest in the red regions, where temperature is higher than the average temperature by 113 K. The map was colored based on ref. 6.

**Table 1:** Elastic molduli and density of bridgmanite at ambient conditions.

	*C*_11_	*C*_22_	*C*_33_	*C*_44_	*C*_55_	*C*_66_	*C*_12_	*C*_13_	*C*_23_	*K*_S_	*G*	*V*_B_	*V*_S_	*ρ*
IXS^a^	450 (7)	498 (9)	420 (8)	190 (6)	168 (5)	148 (4)	117 (8)	125 (8)	137 (9)	236 (4)	166 (2)	7.58 (6)	6.37 (4)	4.103
IXS^b^	448 (6)	519 (5)	438 (5)	178 (3)	180 (4)	138 (3)	124 (5)	113 (8)	160 (5)	244 (3)	165 (1)	7.66 (5)	6.32 (2)	4.140
BLS^17^	515 (5)	525 (5)	435 (5)	179 (4)	202 (3)	175 (4)	117 (5)	117 (5)	139 (6)	246.4	184.2	7.74	6.69	4.108^c^
BLS^18^	482 (4)	537 (3)	485 (5)	204 (2)	186 (2)	147 (3)	144 (6)	147 (6)	146 (7)	264.0	177.3	8.02	6.57	4.108^c^
BLS^19^	481 (4)	528 (3)	456 (4)	200 (2)	182 (2)	147 (2)	125 (3)	139 (3)	146 (3)	253 (3)	175 (2)	7.84	6.52	4.112^c^
LDA^15^	491	554	474	203	176	153	134	139	152	263	178	7.94	6.53	4.174^c^
	488	543	469	193	173	135	147	148	164	268	168	7.74	6.13	4.471^d^
GGA^22^	438	488	422	182	163	134	118	122	136	233	160	7.65	6.34	3.985^c^
GGA-DFPT^23^	494 (2)	511 (2)	426 (2)	193 (1)	176 (1)	151 (1)	109 (1)	137 (1)	149 (1)	247	172	7.88	6.58	3.973^c^
RUS^20^	—	—	—	—	—	—	—	—	—	238	174	7.61	6.51	4.106^c^
UI^21^	—	—	—	—	—	—	—	—	—	247	176	7.75	6.54	4.110^c^
	—	—	—	—	—	—	—	—	—	236	174	7.53	6.47	4.161^e^

The units are GPa, km/s, and g/cm^3^ for elastic moduli, velocities and density, respectively.

^a^Present study (Mg-Bdg).

^b^Present study ((Fe,Al)-Bdg).

^c^MgSiO_3_.

^d^Mg_0.75_Fe_0.25_SiO_3_.

^e^Mg_0.95_Fe_0.05_SiO_3._

**Table 2:** Parameters for elastic wave velocity modeling.

d*V*_B_/dFe [km/s]^a,^*	5.534
d*V*_S_/dFe [km/s]^a,^*	1.040
d*V*_B_/dAl [km/s]^b,^*	−0.024
d*V*_S_/dAl [km/s]^b,^*	−4.465
d*K*_S_/d*T* [GPa/K]^c^	−0.021
d*G*/d*T* [GPa/K]^c^	−0.028
*a* [10^−5^ 1/K]^c^	1.98
*b* [10^−8^ 1/K^2^]^c^	0.80

^a^From the present results and BLS^17^,^18^,^19^,^25^.

^b^From BLS^17^,^18^,^19^,^25^.

^c^From Li^60^ (Thermal expansion coefficient is 
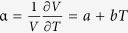
).

^*^Per cation fraction in mole.

**Table 3:** Elastic moduli and density of Mg-Bdg at ambient conditions determined by IXS. The units are the same as those in Table 1.

	*C*_11_	*C*_22_	*C*_33_	*C*_44_	*C*_55_	*C*_66_	*C*_12_	*C*_13_	*C*_23_	*K*_S_	*G*	*V*_B_	*V*_S_	*ρ*
BL35XU	445 (6)	489 (9)	417 (6)	187 (4)	159 (3)	148 (5)	117 (8)	122 (5)	137 (7)	233 (3)	163 (2)	7.54 (5)	6.30 (4)	4.103
BL43LXU	452 (4)	502 (4)	428 (6)	185 (4)	181 (4)	142 (2)	122 (4)	127 (6)	140 (6)	240 (2)	167 (1)	7.64 (4)	6.38 (3)	4.103
Whole data	450 (7)	498 (9)	420 (8)	190 (6)	168 (5)	148 (4)	117 (8)	125 (8)	137 (9)	236 (4)	166 (2)	7.58 (6)	6.37 (4)	4.103
